# Influenza With and Without Oseltamivir Treatment and Neuropsychiatric Events Among Children and Adolescents

**DOI:** 10.1001/jamaneurol.2025.1995

**Published:** 2025-08-04

**Authors:** James W. Antoon, Derek J. Williams, Jean Bruce, Mert Sekmen, Yuwei Zhu, Carlos G. Grijalva

**Affiliations:** 1 Division of Hospital Medicine, Department of Pediatrics, Vanderbilt University Medical Center, Nashville, Tennessee; 2 Division of Pharmacoepidemiology, Departments of Health Policy and Biomedical Informatics, Vanderbilt University Medical Center, Nashville, Tennessee; 3 Department of Biostatistics, Vanderbilt University Medical Center, Nashville, Tennessee

## Abstract

**Importance:**

Reports of pediatric neuropsychiatric events during influenza treatment with oseltamivir have prompted public concerns. However, whether oseltamivir or influenza infection is associated with increased risk of neuropsychiatric events remains unclear.

**Objective:**

To determine the association between influenza, oseltamivir, and serious neuropsychiatric events.

**Design, Setting, and Participants:**

This retrospective cohort study was conducted in a population-based ambulatory setting during the 2016 to 2017 and 2019 to 2020 influenza seasons. Follow-up began on the first day of the influenza season and continued through the earliest occurrence of an outcome event, loss of enrollment, death, age 18 years, or end of the season or study. Children aged 5 to 17 years enrolled in Tennessee Medicaid were for eligible for inclusion. Data analysis was completed from July 2023 to March 2025.

**Exposures:**

Each person-day of follow-up was assigned to 1 of the following 5 mutually exclusive exposure groups: (1) untreated influenza; (2) treated influenza; (3) posttreatment period (period between oseltamivir completion and end of influenza period); (4) influenza prophylaxis; and (5) no exposure.

**Main Outcomes and Measures:**

The primary outcome was a neuropsychiatric event requiring hospitalization, and events were identified using a validated algorithm. Poisson regression estimated incidence rate ratios (IRRs) while accounting for relevant covariates measured on each person-day. Sensitivity analyses examined robustness of findings to alternate exposure and outcome definitions, time-varying outcome risk, negative control outcome, and unmeasured confounding.

**Results:**

Among 692 975 eligible children, a total of 692 295 children (median [IQR] age, 11 [7-14] years; 50.3% female) experienced 1230 serious neuropsychiatric events (898 neurologic and 332 psychiatric) during 19 688 320 person-weeks of follow-up. Among the 151 401 influenza episodes, 66.7% (95% CI, 66.5%-67.0%) were dispensed oseltamivir (60.1% [95% CI, 59.6%-60.6%] among those at high risk for influenza complications). The most common events overall were mood disorders (36.3%) and suicidal or self-harm behaviors (34.2%). Compared with untreated influenza, event rates were lower during oseltamivir-treated influenza periods (IRR, 0.53; 95% CI, 0.33-0.88) and posttreatment periods (IRR, 0.42; 95% CI, 0.24-0.74). Subanalyses suggest that this finding is driven more by a reduction in neurologic events (IRR, 0.45; 95% CI, 0.25-0.82) than psychiatric events (IRR, 0.80; 95% CI, 0.34-1.88). Sensitivity analyses suggest misclassification or unmeasured confounding would not explain these findings.

**Conclusions and Relevance:**

In this cohort study, oseltamivir treatment during influenza episodes was associated with a reduced risk of serious neuropsychiatric events. These findings support oseltamivir use for prevention of these influenza-related complications.

## Key Points

**Question:**

Is the use of the influenza antiviral oseltamivir associated with serious neuropsychiatric events?

**Findings:**

In this cohort study, the risk of serious neuropsychiatric events was lowest during periods without influenza. During influenza periods, treatment with oseltamivir was associated with a reduced risk of serious neuropsychiatric events compared with influenza periods without oseltamivir treatment.

**Meaning:**

Oseltamivir use was associated with a reduced risk of serious neuropsychiatric events when used for influenza treatment; findings from this study should inform both caregivers and clinicians on the safety of oseltamivir and its role in preventing influenza-associated complications.

## Teaser

This cohort study conducted among children enrolled in Tennessee Medicaid determines the association between influenza, oseltamivir, and serious neuropsychiatric events.

## Introduction

Oseltamivir is the most commonly prescribed influenza antiviral in children and adults.^1,2^ When used early, it reduces duration of symptoms, influenza-associated complications, and transmission of influenza.^3,4,5,6,7,8^ In 2006, postmarketing surveillance suggested an increase in serious neuropsychiatric events among children using oseltamivir, prompting the US Food and Drug Administration to change oseltamivir’s warning label.^9^ It is important to note that these warnings were placed on the basis of case reports rather than studies on associated risks for these events.

Most reported oseltamivir-related events have been rapid in onset and resolution, typically within a day of starting and stopping oseltamivir, respectively.^10^ This timing of events is supported by oseltamivir’s short half-life (6-10 hours), low penetration into the central nervous system, and rapid transport out of the central nervous system by the P-glycoprotein transport system.^11,12,13^ Whether oseltamivir is truly associated with neuropsychiatric events remains unclear. Influenza infection itself is associated with encephalitis, seizures, altered mental status, and other neuropsychiatric events.^14,15,16^

Randomized studies do not support an association between oseltamivir treatment and risk of neuropsychiatric events, although aggregated evidence from prophylactic trials has reported increases in neuropsychiatric events among adults.^17^ Observational studies yielded conflicting results^18,19,20,21,22,23,24,25^ and were limited by lack of a validated outcome measure, inclusion of distal events up to 28 days after oseltamivir discontinuation, and difficulty accounting for underlying influenza infection, underlying neuropsychiatric disease, or health care–seeking behaviors.

This cohort study was designed to overcome those limitations and sought to determine associations between oseltamivir use and serious neuropsychiatric events in children and adolescents with and without influenza to better characterize oseltamivir’s risk-benefit profile and inform clinical practice.

## Methods

### Study Design

We assembled a retrospective cohort of children and adolescents aged 5 to 17 years enrolled in Tennessee Medicaid between July 1, 2016, and June 30, 2020 (eFigure 1 in Supplement 1). Tennessee Medicaid databases contain administrative, pharmacy, and health care use data for enrolled children, encompassing approximately half of Tennessee’s children. Data linkages to vital statistics and state hospitalization registries provide a robust longitudinal data source for calculating population-based incidence and examining medication safety and effectiveness.^26,27,28,29^ This study was approved by the Vanderbilt University Medical Center and Tennessee Department of Health institutional review boards and the Division of TennCare with an exemption of informed consent due to the analysis of deidentified data. This study followed the Strengthening the Reporting of Observational Studies in Epidemiology (STROBE) reporting guidelines.^30^

### Influenza Season and Surveillance Periods

Similar to previous studies,^14,31^ the frequency of influenza cases was obtained from the US Centers for Disease Control and Prevention’s FluView surveillance system, and each influenza season was defined as the 13 consecutive calendar weeks containing the maximum frequency of laboratory-confirmed influenza cases in Tennessee.^32^ This approach reduces misclassification of influenza exposure, since the predictive value of influenza diagnoses is directly related to the prevalence of influenza.^33^

Person-time accrued for each participant beginning on the first day of the influenza season and continuing through the earliest occurrence of an incident neuropsychiatric outcome event (see definition to follow), loss of enrollment, death, age 18 years, end of the season, or end of the study. Individuals fulfilling inclusion criteria across multiple seasons accrued person-time within each season and across seasons but could only contribute a single outcome event if it occurred—that is, an individual could no longer contribute person-time to the study after the occurrence of an incident outcome event. Outcome events occurring on the same day as an influenza diagnosis were included in the analysis. Characterization of study exposures and covariates was conducted throughout follow-up for all individuals, began at cohort entry, and was measured at the person-day level, allowing exposures and covariates to change over time. Importantly, follow-up for all influenza episodes (both treated and untreated) began on the same date (ie, the date of influenza diagnosis). Therefore, ascertainment of both exposure and outcome began on the same date for all influenza episodes.

### Exposures

Each person-day of follow-up was assigned to 1 of the following 5 mutually exclusive groups with potential for varying outcome risk (eFigure 2 in Supplement 1): (1) untreated influenza (up to 10 days following diagnosis); (2) treated influenza (days with oseltamivir dispensed during 10-day influenza period); (3) posttreatment period (period between completion of oseltamivir and end of 10-day influenza period); (4) influenza prophylaxis (oseltamivir without influenza); and (5) no exposure (no influenza or oseltamivir exposures). The primary comparison for the study was treated influenza compared to untreated influenza (reference). During the study period, a child could contribute person-time to more than 1 exposure group (eFigure 2 in Supplement 1).

Influenza cases were identified using outpatient *International Statistical Classification of Diseases and Related Health Problems, Tenth Revision (ICD-10)* diagnosis codes (clinic, urgent care, and emergency department [ED]) for influenza, as laboratory results are not available in the databases.^14,33,34,35^ These codes have high positive predictive value (PPV, >85%) in identifying laboratory-confirmed influenza in outpatient settings.^33,35^ Influenza exposure was defined as the period starting on the day of influenza diagnosis (day 0) and continuing through 9 additional days following diagnosis (ie, 10 total days of influenza). Oseltamivir exposure was defined using pharmacy dispensing data and began on the first day of dispensing and continued through the end of days of supply (usually 5 days for influenza treatment and 7-10 days for prophylaxis). Since influenza is typically a 7-day to 10-day illness and oseltamivir treatment is a 5-day course, there is a discrete time period between the end of oseltamivir treatment and the expected resolution of influenza illness. Given that this period may have a differential risk for the outcome, we evaluated this posttreatment influenza period as a separate exposure. To remove potential for immeasurable time bias,^36^ and since detailed in-hospital medication use is not captured by our databases, person-time in the hospital (for nonoutcome reasons) was excluded.^29,37^

### Outcomes

Incident serious neuropsychiatric events were defined as a hospitalization that included an *ICD-10* diagnosis for 1 or more neuropsychiatric events. These events were identified using a validated coding algorithm,^14,38^ which takes into account the position of the coded diagnoses in combination with exclusionary concurrent conditions and is highly accurate (PPV, approximately 90%) in identifying neuropsychiatric events resulting in hospitalizations compared with physician-adjudicated neuropsychiatric events directly related to hospitalization.^38^ The outcome definition includes both neurologic events (seizures, encephalitis, altered mental status, ataxia or movement disorders, vision changes, dizziness, headache, sleeping disorders) and psychiatric events (suicidal or self-harm behaviors, mood disorders, psychosis or hallucination) (eTable 1 in Supplement 1).

### Statistical Analysis

Adjusted incidence rate ratios (aIRRs) with 95% confidence intervals were estimated using multivariable Poisson regression models with robust standard errors clustered at the individual level to account for individuals contributing to multiple exposure groups. Covariates were determined for each person-day of follow-up using a 365-day lookback period and included age; sex; race or ethnicity; influenza season; neurologic and psychiatric comorbid conditions (including prior neurologic or psychiatric hospitalizations); risk factors for influenza complications, including chronic pulmonary (including asthma), cardiovascular, kidney, hepatic, hematologic, metabolic (including obesity), and immunosuppressive diseases^39,40^; ibuprofen or acetaminophen dispensing claims within 2 days of influenza diagnosis; and counts of well-child visits, outpatient sick visits, ED visits, and hospitalizations. Similarly, history of neurologic and psychiatric medications use was assigned for each person-day as described previously. Race and ethnicity constructs were included because of their associations with health care access or health care–seeking behavior, health outcomes, and socioeconomic factors.^41,42^

To complement findings from primary analyses, 4 ad hoc secondary analyses were performed. First, we obtained separate estimates by neurologic or psychiatric category. Second, previous validation studies of the outcome definition demonstrate higher PPV (>98%) when excluding events identified only through secondary diagnoses. Therefore, we obtained separate estimates using 2 alternate outcome definitions focused on events with a primary discharge diagnosis of neuropsychiatric event. Third, given that some events have unclear significance^38^ and are common among medications (headache, dizziness, sleeping disorders, vision changes), we obtained estimates excluding these events from the outcome definition. Fourth, although antivirals are commonly prescribed at the time of diagnosis, some patients may delay using antivirals for a few days after diagnosis. To address potential misclassification of timing of antiviral exposure, we performed an analysis excluding treated episodes that did not have oseltamivir dispensed on the same day as influenza diagnosis.

Planned sensitivity analyses examined study assumptions and robustness of findings. We evaluated 4 alternate exposure definitions (eMethods in Supplement 1). We also performed planned assessments modifying our statistical approach. First, because race and ethnicity designations are social constructs with variable accuracy in electronic health records,^43^ we repeated the Poisson regression excluding race and ethnicity. Second, although the exposure periods of main interest are short in duration, the Poisson regression assumes stable outcome risk during those periods. To evaluate for time-varying outcome risk, we performed a separate time-varying Cox proportional hazards regression comparing treated influenza to untreated influenza accounting for the same covariates included in the main analyses. Third, it is possible that following an influenza diagnosis or oseltamivir dispensing, a mild neuropsychiatric event may occur, generating a new outpatient neuropsychiatric diagnosis or medication prior to a neuropsychiatric hospitalization outcome event. To account for this hypothetical scenario, we repeated the Cox regression only including covariates obtained up to the day of influenza diagnosis (daily covariates during the follow-up period were excluded). Fourth, while most cases of influenza are diagnosed in the outpatient setting or among those discharged from the ED, some individuals may be diagnosed with influenza in the ED and directly admitted to the hospital. We performed a sensitivity analysis excluding cases diagnosed in the ED and directly admitted to the hospital to ensure all individuals had an opportunity to obtain oseltamivir if prescribed. Similarly, we performed an analysis accounting for location of care (ambulatory vs ED) in the regression analysis. Finally, we assessed the potential influence of unmeasured confounding on our findings using E-values and a negative control outcome (hospitalization for an acute appendicitis) analysis^44,45^ (see the eMethods in Supplement 1 for details). Analyses were performed using Stata/MP version 18.0 (StataCorp) and R version 4.1.1 (R Foundation).

## Results

### Characteristics of the Study Cohort

**Figure 1.**
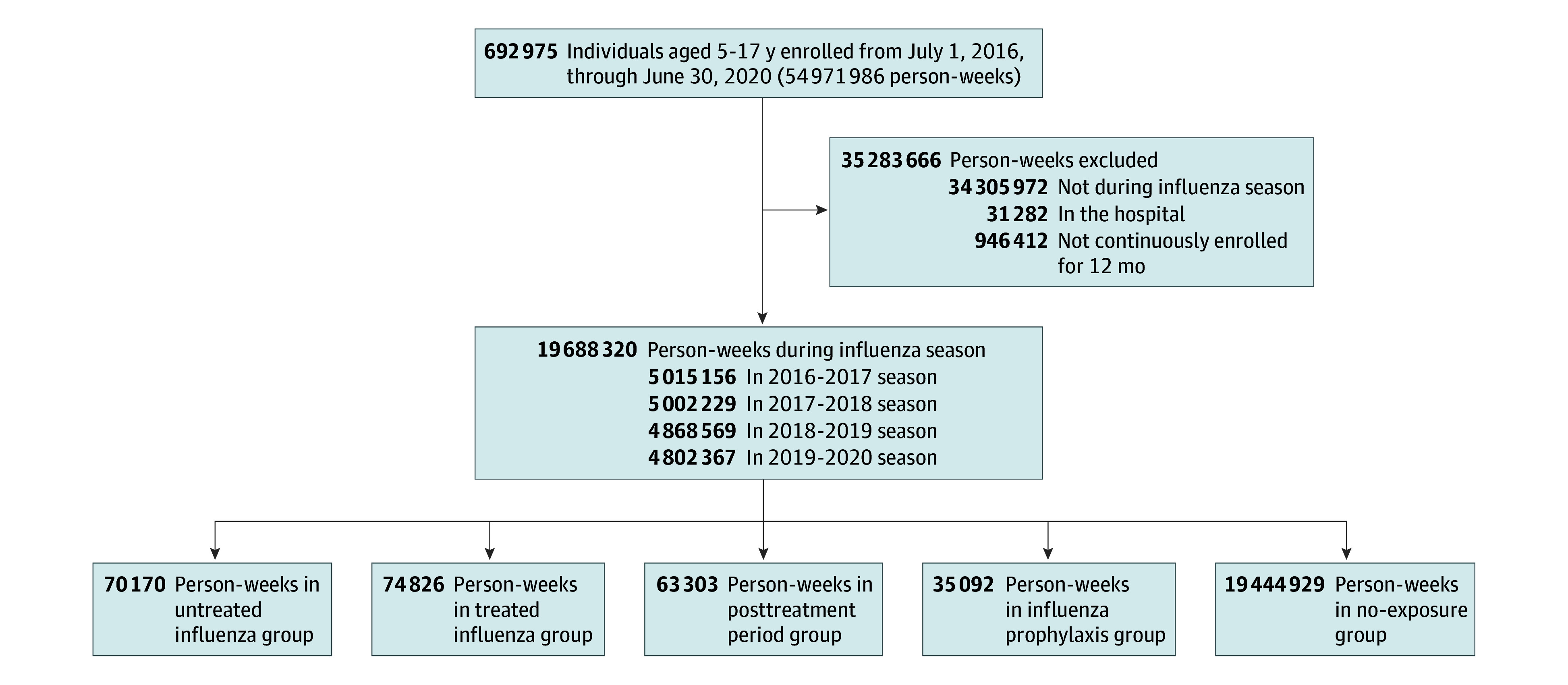
Flow Diagram

The cohort comprised 692 295 children (median [IQR] age, 11 [7-14] years; 50.3% female) and accrued 1230 serious neuropsychiatric events (898 neurologic, 332 psychiatric) during 19 688 320 person-weeks of follow-up (Figure 1). Clinical characteristics were generally similar across exposure groups (Table). There were 129 134 unique individuals who experienced 151 401 influenza episodes. Less than 2% of these individuals had more than 2 episodes of influenza during the study period. Among those with an influenza episode, 66.7% (95% CI, 66.5%-67.0%) were dispensed oseltamivir. Of those, 88.9% (95% CI, 88.7%-89.1%) were dispensed oseltamivir on the same day of influenza diagnosis. Among those at high risk for influenza complications, 60.1% (95% CI, 59.6%-60.6%) received an antiviral. There were 31 out-of-hospital deaths (30 in the no-exposure group). The proportions of censoring for loss of enrollment and age greater than 18 years were low and similar across exposure groups (eTable 2 in Supplement 1).

**Table.** Characteristics of the Cohort According to Exposure Assignment

Characteristic	%					
	Total	Exposure cohort				
		No exposure	Untreated influenza	Treated influenza	Posttreatment period	Influenza prophylaxis
Person-weeks, No. (%)	19 688 320 (100)	19 444 929 (98.8)	70 170 (0.4)	74 826 (0.4)	63 303 (0.3)	35 092 (0.2)
Age, median (IQR), y	11 (7-14)	11 (7-14)	9 (6-12)	9 (6-12)	9 (7-12)	10 (7-14)
Age category, y						
5-11	56.3	56.6	72.1	71.4	68.7	60.9
12-17	43.7	44.4	27.9	28.6	31.3	39.1
Race or ethnicity^a^						
American Indian	0.2	0.2	0.2	0.2	0.2	0.2
Asian	0.1	0.1	0.1	0.1	0.1	0.1
Black	22.0	22.0	18.0	14.4	14.1	12.7
Hispanic/Latino	0.2	0.2	0.1	0.2	0.2	0.1
Southeast Asian	0.9	0.9	0.9	1.0	1.0	0.8
White	53.2	53.10	59.3	62.8	63.1	63.6
Other or unknown^b^	23.4	23.5	21.4	21.3	24.3	22.5
Sex						
Female	50.3	50.3	50.5	51.4	51.4	49.0
Male	49.7	49.7	49.5	48.6	48.6	51.0
Risk factor for influenza complications^c^						
Any	24.8	24.8	23.7	26.5	26.9	28.8
Asthma	9.3	9.3	10.4	12.4	12.5	12.0
Obesity	10.7	10.7	9.5	10.0	10.3	10.0
Non-neurologic comorbidity	7.2	7.2	6.6	7.6	7.7	10.8
Neuropsychiatric comorbidity						
Any	30.3	30.4	25.9	27.7	27.7	32.6
Neurologic comorbidity	2.8	2.8	2.1	2.3	2.3	3.7
Psychiatric comorbidity	29.5	29.6	25.2	27.0	27.1	31.7
Concurrent medications						
Antipyretic medications	0.1	0.0	5.6	6.7	6.5	0.0
Neurologic medications	2.2	2.2	1.4	1.5	1.6	2.9
Psychiatric medications	11.6	11.7	8.8	11.0	10.7	14.0
Influenza season						
2016-2017	25.5	25.6	15.3	17.4	17.7	16.3
2017-2018	25.4	25.4	24.2	28.0	28.1	35.2
2018-2019	24.7	24.7	24.8	25.7	24.7	22.0
2019-2020	24.4	24.3	35.6	28.9	29.6	26.4
Health care utilization in prior year, median (IQR), No.						
Well visits	1 (0-1)	1 (0-1)	1 (0-1)	1 (0-1)	1 (0-1)	1 (0-1)
Sick visits	3 (1-6)	3 (1-6)	4 (2-7)	4 (2-8)	4 (2-8)	5 (2-8)
ED visits	0 (0-1)	0 (0-1)	1 (0-2)	0 (0-1)	0 (0-1)	0 (0-1)
Hospitalizations	0 (0-0)	0 (0-0)	0 (0-0)	0 (0-0)	0 (0-0)	0 (0-0)

Abbreviation: ED, emergency department.

^a^ Race and ethnicity were ascertained by self-report and are reported to describe the study population.

^b^ Other included those reporting their race or ethnicity as Cuban, Haitian, or other race or ethnicity.

^c^ Excludes neurologic conditions, pregnant individuals, and those with long-term aspirin use.

### Serious Neuropsychiatric Events

The most common serious neuropsychiatric events overall were mood disorders (36.3%) and suicidal or self-harm behaviors (34.2%) followed by seizures (13.7%) (eTable 3 in Supplement 1). Seizures (34.1%) were the most common events during influenza exposure periods, whereas mood disorders and suicidal or self-harm behaviors were noted in 20.8% and 14.5% of influenza exposure periods, respectively. Among those experiencing an event during an influenza exposure period, the median (IQR) intervals between the influenza diagnosis and the occurrence of a serious neuropsychiatric event were 1 (1-2) days for untreated influenza and 2.5 (1-4) days for treated influenza (eFigure 3 in Supplement 1). Among those with an influenza diagnosis, 76.7% of events occurred within 5 days of influenza diagnosis (92.8% in the untreated influenza group).

### Oseltamivir Use and Neuropsychiatric Events

**Figure 2.**
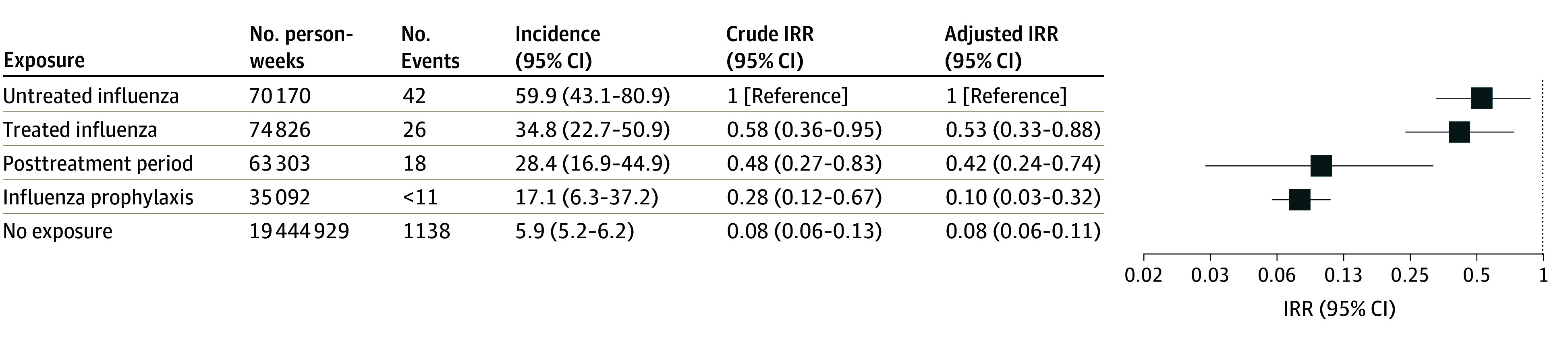
Forest Plot of Incidence Rate Ratios (IRRs) for Serious Neuropsychiatric Events Reporting of cells with values <11 was restricted to ensure confidentiality. Incidence is expressed per 100 000 person-weeks.

The overall incidence of serious neuropsychiatric events was 6.25 (95% CI, 5.90-6.61) per 100 000 person-weeks. In multivariable analyses, the risk of serious neuropsychiatric events during influenza exposure periods was lower in those treated with oseltamivir (treated influenza: aIRR, 0.53; 95% CI, 0.33-0.88; posttreatment period: aIRR, 0.42; 95% CI, 0.24-0.76) compared to untreated influenza (Figure 2). Relative risks were also low during periods with neither influenza nor oseltamivir dispensing (aIRR, 0.08; 95% CI, 0.06-0.11) and oseltamivir prophylaxis periods (aIRR, 0.10; 95% CI, 0.03-0.32) compared to untreated influenza. Secondary analyses examining neurologic and psychiatric events separately yielded aIRR estimates for the treated influenza group of 0.45 (95% CI, 0.25-0.83) and 0.80 (95% CI, 0.34-1.88), respectively.

### Sensitivity Analyses

**Figure 3.**
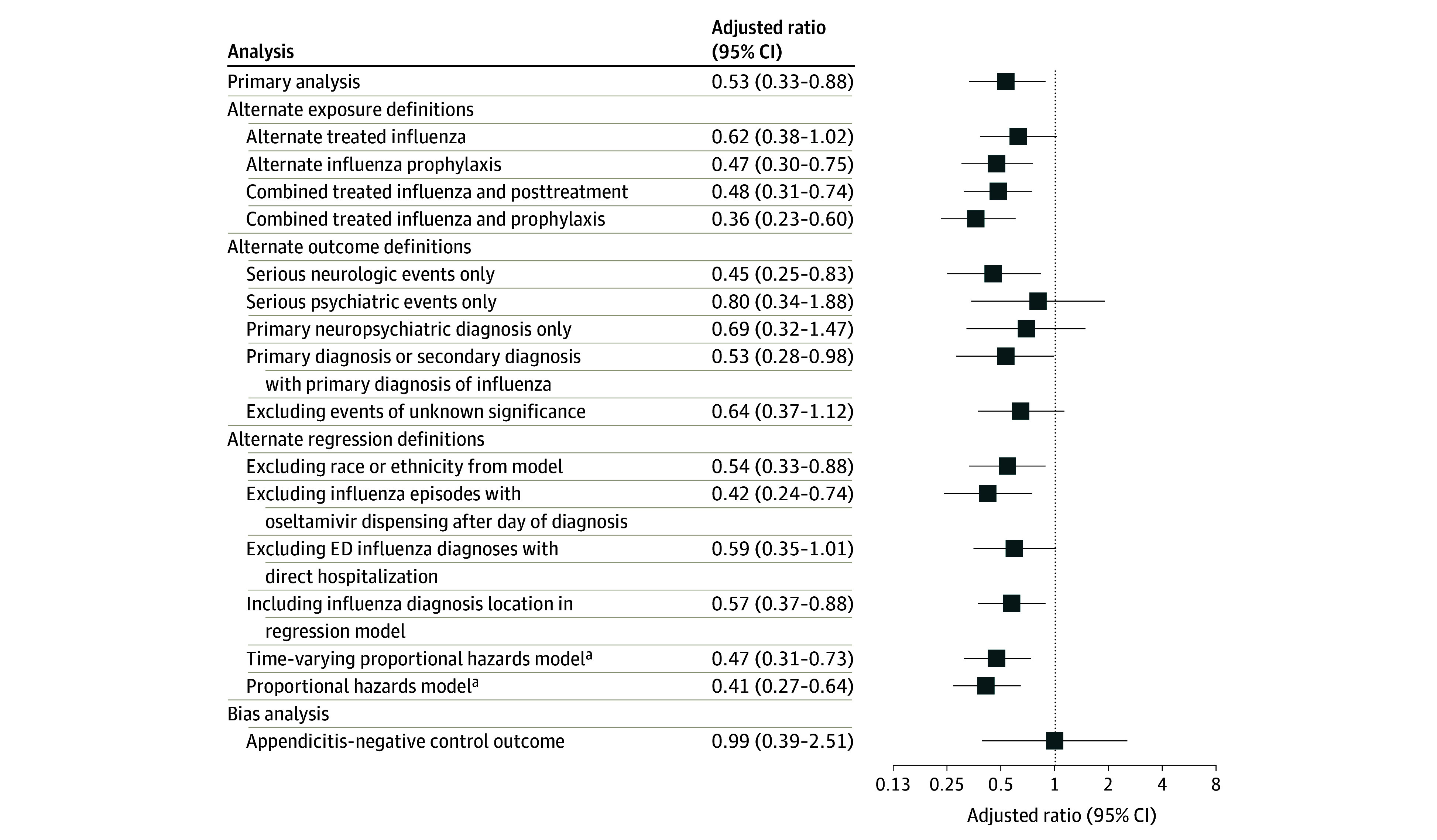
Forest Plot of Measures of Association for Serious Neuropsychiatric Events Resulting From Alternate Analyses Unless otherwise specified, adjusted ratio values represent incidence rate ratios. The alternative exposure definitions varying the specificity and sensitivity of the exposure assignment were (1) alternate treated influenza, defined as influenza diagnosis and oseltamivir supply ≤5 days; (2) alternate influenza prophylaxis, defined as no influenza diagnosis and oseltamivir ≥7 days; (3) treated influenza, defined as treated influenza or posttreatment period (combined); and (4) treated influenza, defined as treated influenza or influenza prophylaxis (combining all oseltamivir exposures). The alternate outcome definitions were (1) serious neurologic event only (excludes psychiatric events); (2) serious psychiatric events only (excludes neurologic events); (3) primary neuropsychiatric diagnosis only (excludes cases of secondary diagnosis of neuropsychiatric event without a primary neuropsychiatric diagnosis); (4) primary diagnosis of a neuropsychiatric events or secondary diagnosis of a neuropsychiatric event with a concurrent primary diagnosis of influenza; and (5) excluding events of unknown significance (headaches, dizziness, vision changes, and sleep disorders). ED indicates emergency department. ^a^Indicates adjusted hazard ratio.

**Figure 4.**
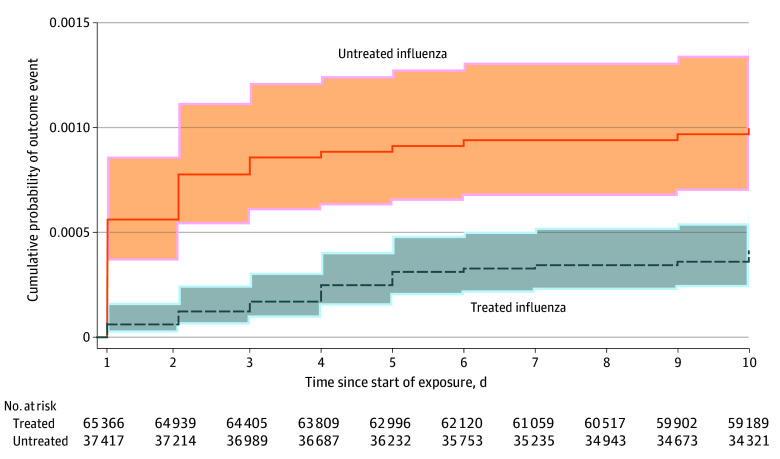
Cumulative Incidence of Serious Neuropsychiatric Events Shaded areas represent 95% confidence intervals.

Planned analyses evaluating the robustness of study findings are summarized in Figure 3. Varying exposure (eTable 4 in Supplement 1) or outcome (eTable 5 in Supplement 1) definitions, excluding race or ethnicity (eTable 6 in Supplement 1) from the primary regression, excluding episodes of treated influenza with dispensing after the day of influenza diagnosis, and excluding cases of influenza diagnoses in the ED with direct admission to the hospital yielded similar results to those from the primary analysis. An alternative proportional hazards model incorporating time-varying outcome risk or excluding covariates during the follow-up period each also yielded similar results to the primary analyses (Figure 3 and Figure 4). The measured E-value was 3.2, suggesting unmeasured confounders would need at least a 3.2-fold magnitude of association with both oseltamivir and serious neuropsychiatric events to shift the observed IRR for treated influenza to the null (eFigure 4 in Supplement 1). Finally, there was no association observed between treated influenza and appendicitis, the negative control outcome (eTable 7 in Supplement 1).

## Discussion

In this cohort study, we evaluated whether oseltamivir use was associated with the risk of serious neuropsychiatric events in a state Medicaid population of children and adolescents with approximately 20 million person-weeks of follow-up over 4 influenza seasons using validated definitions to precisely characterize exposures and outcomes and accurately estimate their associations. Summarizing our findings, risk of serious neuropsychiatric events increased during periods of influenza infection. During these periods, treatment with oseltamivir was associated with an approximately 50% reduction in the incidence of serious neuropsychiatric events compared with influenza periods without oseltamivir treatment. These results support the dual assertions that influenza infection, independent of other measured factors, increases the risk of serious neuropsychiatric events and that treatment with oseltamivir may protect against these influenza-related complications.

Previous meta-analyses of clinical trials evaluating oseltamivir and neuropsychiatric events had mixed findings. A Cochrane review of adult trials, as well as subsequent meta-analyses of trials in children and adults with influenza, did not find an association between oseltamivir treatment and neuropsychiatric events, although events were rare and estimates imprecise.^3,5,17^ A secondary analysis combining periods receiving and not receiving prophylactics in adults without influenza, however, suggested an increased risk of psychiatric events in the Cochrane study.^17^ Among the 4 included prophylactic trials, 2 were among older adults and only 1 included a pediatric population. Importantly, influenza infection itself could increase the risk of neuropsychiatric events,^15,46,47^ so distinguishing treatment from prophylaxis populations is warranted. Furthermore, psychiatric events were not the primary end point of the included trials, and the review acknowledged high risk for attrition and reporting bias resulting from incomplete outcome data and lack of standardized definitions for adverse events, which included subjective events, such as “alcohol related” and “nervousness.” Importantly, given the short half-life of oseltamivir,^12,13^ inclusion of reported events up to 18 days after stopping oseltamivir may have led to misattribution of neuropsychiatric events.

Our study found a reduction in overall neuropsychiatric events during influenza episodes treated with oseltamivir compared to untreated influenza episodes. Evaluation of the timing of these events suggests biological activity of oseltamivir related to these events, with outcome events in the treated influenza groups generally occurring less often and later in the influenza episode compared to untreated influenza. Subanalyses suggest that this finding is driven more by a reduction in neurologic events than psychiatric events. While seizures were the most common neurologic event and likely main driver of the effect estimate, there was also a reduction in the frequency of nonseizure neurologic events in the treated group. While the point estimate for psychiatric events was less than 1, the low number of outcomes resulted in imprecise confidence intervals that crossed the null. However, the findings of these subanalyses do not support the assertion that oseltamivir is associated with an increased risk of psychiatric events.

In the absence of new data from large randomized clinical trials, observational studies can contribute evidence to complement discussions about the safety of oseltamivir. Among 7 observational pediatric studies, 3 found that oseltamivir was associated with decreased risk of neuropsychiatric events, similar to our study,^18,19,24^ while 2 each demonstrated no association^20,23^ or increased risk associated with oseltamivir.^21,22^ Blumental and colleagues^18^ reported a similar reduction of neuropsychiatric events associated with oseltamivir use (adjusted odds ratio, 0.68; 95% CI, 0.67, 0.90) in the adolescent population, and theirs is the only study that examined serious events leading to hospitalization within 14 days of diagnosis while controlling for prior health care utilization.^18^ A study by Huh and colleagues^23^ did not identify associations between oseltamivir and neuropsychiatric events, although outcome events were primarily represented by outpatient encounters for dizziness (70%) or anxiety (11%), and a secondary analysis excluding many of these events suggested a modest protective effect of oseltamivir. A self-controlled case-crossover study^21^ reported an increased risk of neuropsychiatric events with oseltamivir treatment, although that analysis used control windows immediately prior to hazard periods, a design that makes accurate comparisons of untreated and treated influenza challenging.^42,43^ Another study reported an increased risk in adolescents 10 to 19 years old but not among those younger than 9 years within 5 days of oseltamivir prescribing.^22^ Most events were outpatient neuropsychiatric events. Differences in exposure and outcome definitions, underlying study populations, and methodological decisions and limitations likely contributed to the differing results. Strengths of our study include precise exposure assignment at the person-day level, use of more accurate and validated *ICD-10* influenza codes, use of a validated outcome definition, evaluation of a negative control outcome, and a robust set of sensitivity analyses.

We implemented multiple strategies to minimize misclassification and confounding in this study. To minimize misclassification, we used validated measures to identify influenza cases and neuropsychiatric events and used a strict influenza season definition to focus on periods with known influenza activity. Measured clinical and sociodemographic characteristics were similar across those with untreated and treated influenza, and our analyses accounted for important known risk factors for serious neuropsychiatric events, including underlying neurologic and psychiatric conditions and measures of health care utilization. We also accounted for measured antipyretics use, since these drugs may affect the risk of fever-related delirium or hallucinations.^48^ Furthermore, to minimize confounding by severity (eg, more severe influenza increasing the likelihood of both oseltamivir treatment and neuropsychiatric event hospitalizations), we restricted our influenza definition to outpatient and ED diagnoses. An analysis excluding cases of diagnosis in the ED with direct hospitalization (without being discharged from ED) and, separately, accounting for location of influenza diagnosis did not alter the study findings. Estimates excluding covariates obtained during the follow-up period and separately incorporating time-varying risk for the outcome yielded results nearly identical to the primary analyses. Results from other planned sensitivity analyses, including alternate exposure and outcome definitions, E-value estimation, and a negative control analysis, suggest that unmeasured or residual confounding are unlikely to explain the observed associations.

### Limitations

Despite extensive efforts to control confounding, there may be residual differences due to factors like chronic medication adherence or propensity to seek care and/or use antivirals (ie, healthy user biases). Our analyses did not account for influenza strain or vaccination status, as these are not reliably captured in study databases. However, guidelines recommend oseltamivir regardless of strain or vaccination status,^39,49,50^ and neither factor has been associated with outpatient oseltamivir use.^51,52^ Similarly, we could not account for symptom duration from disease onset to the time of influenza diagnosis, and it is possible that those in the treated influenza groups presented earlier in their disease course than the untreated group.^53,54^ Nevertheless, neuropsychiatric events early in the influenza disease course (prior to an influenza diagnosis) would be misclassified into the no-exposure group and bias comparisons toward the null. Including those events into the untreated influenza group would increase the observed association. Information on prescribing was not available in our database, and there may be some individuals prescribed oseltamivir who did not fill the prescription. The study was performed within Tennessee’s Medicaid population, and findings may not be generalizable to other populations. Finally, our study focused on serious neuropsychiatric events that required hospitalization, but we did not evaluate milder events that were identified and managed in the outpatient setting or that did not result in a health care encounter.

## Conclusions

In this population-based cohort of children and adolescents enrolled in Tennessee Medicaid, the risk of serious neuropsychiatric events was lowest during periods without influenza exposure. During influenza exposure periods, treatment with oseltamivir was associated with an approximately 50% reduction in the risk of serious neuropsychiatric events compared to influenza periods without oseltamivir. Findings from this study should inform both caregivers and clinicians on the safety of oseltamivir and its role in preventing influenza-associated complications.

## Notes

Supplement 1. eMethods. Supplementary Methods eTable 1. Serious Neuropsychiatric Event Identification Algorithm eTable 2. Censoring Events Stratified by Exposure Group eTable 3. Frequency Distribution of Neuropsychiatric Events by Category eTable 4. Incidence-Rate Ratios Examining Alternate Exposure Definitions eTable 5. Incidence-Rate Ratios of Ad-Hoc Sub-Analysis Obtaining Estimates for Alternative Outcome Definitions eTable 6. Incidence-Rate Ratios When Excluding Race or Ethnicity From Statistical Model eTable 7. Incidence-Rate Ratios for Negative Control Outcome eFigure 1. General Overview of Study Design and Measurements eFigure 2. Person-Time Assignment Examples eFigure 3. Time Between Start of Exposure and Serious Neuropsychiatric Outcome Event eFigure 4. E-Value Analysis eReferences.

Supplement 2. Data Sharing Statement
