# Supplementary material for: Influenza With and Without Oseltamivir Treatment and Neuropsychiatric Events Among Children and Adolescents
Source: JAMA Neurol. 2025 Aug 4;82(10):1013–21. doi: 10.1001/jamaneurol.2025.1995 (PMC12322824; doi:10.1001/jamaneurol.2025.1995)
Supplement: Supplement 1. — eMethods. Supplementary Methods eTable 1. Serious Neuropsychiatric Event Identification Algorithm eTable 2. Censoring Events Stratified by Exposure Group eTable 3. Frequency Distribution of Neuropsychiatric Events by Category eTable 4. Incidence-Rate Ratios Examining Alternate Exposure Definitions eTable 5. Incidence-Rate Ratios of Ad-Hoc Sub-Analysis Obtaining Estimates for Alternative Outcome Definitions eTable 6. Incidence-Rate Ratios When Excluding Race or Ethnicity From Statistical Model eTable 7. Incidence-Rate Ratios for Negative Control Outcome eFigure 1. General Overview of Study Design and Measurements eFigure 2. Person-Time Assignment Examples eFigure 3. Time Between Start of Exposure and Serious Neuropsychiatric Outcome Event eFigure 4. E-Value Analysis eReferences. [file jamaneurol-e251995-s001.pdf]

Supplemental Online Content

Antoon JW, Williams DJ, Bruce J, Sekmen M, Zhu Y, Grijalva CG. Influenza With and Without Oseltamivir Treatment and Neuropsychiatric Events Among Children and Adolescents. *JAMA Neurol*. Published online August 4, 2025. doi:10.1001/jamaneurol.2025.1995

eMethods. Supplementary Methods

eTable 1. Serious Neuropsychiatric Event Identification Algorithm

eTable 2. Censoring Events Stratified by Exposure Group

eTable 3. Frequency Distribution of Neuropsychiatric Events by Category

eTable 4. Incidence-Rate Ratios Examining Alternate Exposure Definitions

eTable 5. Incidence-Rate Ratios of Ad-Hoc Sub-Analysis Obtaining Estimates for Alternative Outcome Definitions

eTable 6. Incidence-Rate Ratios When Excluding Race or Ethnicity From Statistical Model

eTable 7. Incidence-Rate Ratios for Negative Control Outcome

eFigure 1. General Overview of Study Design and Measurements

eFigure 2. Person-Time Assignment Examples

eFigure 3. Time Between Start of Exposure and Serious Neuropsychiatric Outcome Event

eFigure 4. E-Value Analysis

eReferences.

© 2025 Antoon JW et al. *JAMA Neurology*.

This supplemental material has been provided by the authors to give readers additional information about their work.

© 2025 Antoon JW et al. *JAMA Neurology*.

eMethods. Supplementary Methods

Ascertainment of Influenza Cases and Influenza Season Definition

Outpatient (clinic, emergency department and urgent care) influenza infections were identified using the following ICD-10 diagnoses codes[1,2]:

(J09-Influenza due to identified zoonotic or pandemic influenza virus, J10- Influenza due to identified seasonal influenza virus, J10.0- Influenza with pneumonia, seasonal influenza virus identified, J10.1- Influenza with other respiratory manifestations, seasonal influenza virus identified, J10.8- Influenza with other manifestations, seasonal influenza virus identified, J11- Influenza, virus not identified, J11.0- Influenza with pneumonia, virus not identified, J11.1- Influenza with other respiratory manifestations, virus not identified, J11.8- Influenza with other manifestations, virus not identified

The specificity of influenza diagnosis is directly related to circulating prevalence of the virus.[2-4] When compared with manual review of clinical and laboratory records, these influenza ICD-10 diagnosis codes have high predictive value in identifying influenza illness in children in the outpatient (PPV>85%[5]), emergency department (PPV>90%)[2] and inpatient (PPV>90-95%[5]) settings. To increase the specificity of the influenza diagnosis even further and mitigate the potential for misclassification of influenza diagnoses included in the study, each study influenza season was comprised of the 13 consecutive weeks that contained the maximum number of laboratory-confirmed influenza cases in public health surveillance in the state of Tennessee, based on data obtained from the Centers for Disease Control and Prevention Influenza Activity & Surveillance program.[2,6]

© 2025 Antoon JW et al. *JAMA Neurology*.

Ascertainment of Oseltamivir Exposures

Oseltamivir exposures were identified using Medicaid dispensing claims. Oseltamivir exposure was defined starting on the day of dispensing and continued through the projected exhaustion of the dispensed days of supply. To confirm the proportion of ascertainment of oseltamivir exposure among individuals with an influenza diagnosis, a manual review of 100 random encounters within the study population was performed. Out of 100 individuals with influenza, 67 had a confirmed antiviral dispensing. Among the entire study cohort, 66.9% of individuals with influenza were identified to have an oseltamivir dispensing.

Identification of Underlying Neurologic and Psychiatric Conditions

Underlying neurologic complex chronic conditions were identified using the pediatric complex chronic conditions (CCC) classification system, version 2.[7] Psychiatric co-morbid conditions were identified using the Pediatric Mental Health Disorders Classification System.[8] The presence of an underlying neurologic or psychiatric condition was defined as the presence of two outpatient or one inpatient ICD-10 diagnosis in the previous 365 days. A look-back period of one year for each person-day of exposure was used to identify neurologic or psychiatric co-morbid conditions.

Children at High-risk for Influenza Complications Definition

Children at high risk for influenza complications were identified using the American Academy of Pediatrics[9], Infectious Disease Society of America[10], and Centers for Disease Control and Prevention[11] definitions for high-risk children and included: chronic neurological conditions (neurological conditions defined using the pediatric CCC classification system version 2)[7], asthma diagnosis[12], obesity diagnosis[13], complex chronic conditions (non-

© 2025 Antoon JW et al. *JAMA Neurology*.

neurologic CCCs such as immunosuppressive, cardiovascular, kidney, hepatic, hematologic, metabolic and pulmonary diseases)[7]. Underlying high-risk conditions were defined as the presence of two outpatient or one inpatient ICD-10 diagnosis in the previous 365 days. A look-back period of one year each person-day of exposure was used to identify high-risk co-morbid conditions. Individuals residing in a nursing home or other chronic care facility and those of Indigenous American and Alaska Native race and ethnicity were excluded as these covariates are not reliably captured in the study database. Pregnant individuals and children with long-term aspirin use were excluded due to their low frequency in children.

Ascertainment of Neurologic and Psychiatric Medications

Neurologic and psychiatric medications were identified using pharmacy fills data, including NDC (national drug classification) information, and were assigned at the person-day level and use was assumed from the dispensing date through the exhaustion of the dispensed days of supply. Neurologic medications included barbiturates and anticonvulsants, skeletal muscle relaxant, anti-Parkinson agents, parasympathomimetic ages, tardive dyskinesia agents, and agents used to treat migraines. Psychiatric medications included meprobamate, buspirone hydrochloride, chloral hydrate, hydroxyzine, paraldehyde, ethchlorvynol, zolpidem tartrate, zaleplon, eszopiclone, ramelteon, amitriptyline, perphenazine-amitriptyline, fluoxetine-olanzapine, chlordiazepoxide-amitriptyline, cannabidiol extract, methylphenidate hydrochloride, caffeine, modafinil, dexmethylphenidate hydrochloride, armodafinil, propranolol, atenolol, lithium, guanfacine, clonidine, amphetamine and amphetamine derivatives, tricyclics, MAO inhibitors, miscellaneous antidepressants, selective serotonin reuptake inhibitors, serotonin-norepinephrine reuptake inhibitors, phenothiazines (antipsychotics), butyrophenones (antipsychotics), other antipsychotics, benzodiazepines.

© 2025 Antoon JW et al. *JAMA Neurology*.

Ascertainment of Serious Neuropsychiatric Outcomes

The primary outcome of serious neuropsychiatric events was defined as an event requiring hospitalization and identified using a validated algorithm (Table S1). The algorithm had a positive predictive value of ~90% for identifying neuropsychiatric events that were present on admission to the hospital and directly related to hospitalization as compared to review of clinical records and physician adjudication.[14] Categorization of neuropsychiatric event was originally developed on the basis of consensus expert opinion among a study team of pediatric psychiatrists, general pediatricians, pediatric hospitalists, pharmacoepidemiologists, and complex care pediatricians. Categories include: homicidal, suicidal, or self-harm behaviors, mood disorders (including anxiety and stress), psychosis, hallucination, altered mental status, ataxia or movement disorders, encephalitis or encephalopathy, seizures, dizziness, headache, sleeping disorders, and vision changes.[14]

Ascertainment of Negative Control Outcome of Appendicitis Hospitalization

A negative control outcome of appendicitis hospitalized was defined as a hospitalization with a *ICD-10* diagnosis (K35-K37) or *ICD-10-PCS* code for an appendicitis procedure:, 0D5J0Z3, 0D5J0ZZ, 0D5J3Z3, 0D5J3ZZ, 0D5J4Z3, 0D5J4ZZ, 0D5J7ZZ, 0D5J8ZZ, 0D9J00Z, 0D9J0ZX, 0D9J0ZZ, 0D9J30Z, 0D9J3ZX, 0D9J3ZZ, 0D9J40Z, 0D9J4ZX, 0D9J4ZZ, 0D9J70Z, 0D9J7ZX, 0D9J7ZZ, 0D9J80Z, 0D9J8ZX, 0D9J8ZZ, 0DBJ0ZX, 0DBJ0ZZ, 0DBJ3ZX, 0DBJ3ZZ, 0DBJ4ZX, 0DBJ4ZZ, 0DBJ7ZX, 0DBJ7ZZ, 0DBJ8ZX, 0DBJ8ZZ, 0DCJ0ZZ, 0DCJ3ZZ, 0DCJ4ZZ, 0DCJ7ZZ, 0DCJ8ZZ, 0DDJ3ZX, 0DDJ4ZX, 0DDJ8ZX, 0DFJ0ZZ0, DFJ3ZZ, 0DFJ4ZZ, 0DFJ7ZZ, 0DFJ8ZZ, 0DFJXZ, Z0DNJ0ZZ, 0DNJ3ZZ, 0DNJ4ZZ, 0DNJ7ZZ, 0DNJ8ZZ, 0DQJ0ZZ, 0DQJ3ZZ, 0DQJ4ZZ, 0DQJ7ZZ, 0DQJ8ZZ, 0DTJ0ZZ, 0DTJ4ZZ, 0DTJ7ZZ, 0DTJ8ZZ, 0DTK0ZZ, 0DTK4ZZ

© 2025 Antoon JW et al. *JAMA Neurology*.

Sensitivity Analyses Examining Alternate Exposure Definitions

For sensitivity analyses, alternate definitions of oseltamivir exposure were used:

- Exposure Sensitivity Analysis 1: The IDSA, CDC and AAP recommend a 5-day duration of oseltamivir to treat acute influenza illness. We restricted the treated influenza exposure group to those with an acute treatment duration in concordance with these national guidelines/recommendations. Therefore, in this sensitivity analysis, treated influenza was defined as the overlapping days of an influenza exposure period AND oseltamivir dispensing with days supply of $\leq$ 5.

- Exposure Sensitivity Analysis 2: The IDSA, CDC and AAP recommend a 7-day duration for post-exposure prophylaxis for influenza. We restricted the influenza prophylaxis group to those with the prophylaxis duration of treatment as recommended by national guidelines/recommendations. Therefore, in this sensitivity analysis, influenza prophylaxis defined as the overlapping days of an influenza exposure period AND oseltamivir dispensing with days supply of $\geq$ 7.

- Exposure Sensitivity Analysis 3: To allow identification of outcome events that may have occurred right after completion of oseltamivir treatment, we repeated the analysis combining the treated influenza and post-treatment period groups into the treated influenza groups.

- Exposure Sensitivity Analysis 4: We assessed all oseltamivir exposures together by repeating the analysis combining the treated influenza and influenza prophylaxis groups into the treated influenza group.

Sub-Analyses Examining Alternate Outcome Definitions

To examine alternative outcome definitions, we obtained separate estimates for the following:

© 2025 Antoon JW et al. *JAMA Neurology*.

- Outcome sub-analysis Analysis 1: Although influenza is associated with both neurologic and psychiatric events, a Cochrane review reported an association with psychiatric outcome during prophylactic use in adults. To evaluate the association between oseltamivir use and risk of psychiatric events specifically, we obtained separate estimates for serious neurologic events and serious psychiatric events.

- Outcome sub-analysis 2: Previous validation studies of the neuropsychiatric outcome algorithm demonstrate higher positive predictive value (>98%) when excluding events with secondary diagnosis of a neuropsychiatric alone.[14] However, that study was not restricted to influenza cases. There is variation in hospitalization coding practices and the underlying disorder or cause for the admission is often assigned the primary diagnosis. Therefore, we obtained separate diagnoses for two outcome definitions accounting for these practices. A primary diagnosis was identified as the principal diagnosis in the first position of the hospitalization. Secondary diagnoses were defined as any diagnosis not in the first position. In the Tennessee Medicaid databases, up to 16 diagnoses may be recorded for one hospitalization.

    - A) Defining the outcome as a hospitalization with a primary diagnosis of a neuropsychiatric event while excluding cases with only a secondary diagnosis of a neuropsychiatric event

    - B) Defining the outcome as a hospitalization with a primary diagnosis of a neuropsychiatric event OR a primary diagnosis of influenza with a secondary diagnosis of a neuropsychiatric event

- Outcome sub-analysis 3: Certain neuropsychiatric events included in the outcome definition have unknown clinical significance, may be less clinically relevant than

© 2025 Antoon JW et al. *JAMA Neurology*.

other events, and are common across many different medications. Specifically, these include headache, dizziness, vision changes, and sleep disturbances.[14] Therefore, we obtained estimates excluding these events from the outcome definition.

Sub-analysis – Exclusion of influenza episodes with oseltamivir dispensing after day of diagnosis

Antivirals are most commonly prescribed at the time of diagnosis. However, some patients may delay using antivirals for several after diagnosis. To evaluate potential misclassification of timing of antiviral exposure, we performed an analysis excluding treated episodes that were not dispensed oseltamivir on the same day as influenza diagnosis. In this analysis the primary comparison is between untreated influenza and treated influenza on the day of influenza diagnosis.

Sub Analysis - Time-Varying Cox Proportional Hazard Analysis

To further compare treated influenza to untreated influenza while allowing time-varying risk of serious neuropsychiatric events, we performed a multivariable time-varying Cox proportional hazards regression while accounting for the same set of covariates described for the primary analysis. Similar to the main analysis, model-estimated standard errors were clustered at the individual level to account for individuals contributing to multiple exposure groups and repeat exposures. Follow-up for outcome ascertainment started on the first day of influenza exposure and followed through 10 days or occurrence of a censoring event, whichever occurred first. Each person-day during follow-up was considered a discrete risk period. Because each covariate was assessed each person-day with a separate lookback assessment every day, it is possible for the covariate profile to change during the 10 days follow-up period. Therefore, each covariate was considered as time-varying in the regression model.

© 2025 Antoon JW et al. *JAMA Neurology*.

Sub-analysis - Cox Proportional Hazard Analysis Excluding Covariates Obtained During Follow-up Period

It is possible that following an influenza diagnosis or oseltamivir dispensing, a mild neuropsychiatric event may occur. This event may generate a new outpatient neuropsychiatric diagnosis or medication prior to, and also be related to, a neuropsychiatric hospitalization outcome event. To account for this hypothetical scenario, we compared treated influenza to untreated influenza using a Cox proportional hazards model using the same covariate profile as the primary analysis. In this analysis the covariate profile was ascertained at the time of influenza diagnosis and daily covariates between the day of influenza diagnosis and the end of follow-up were not included in the model. Unadjusted Kaplan Meier failure curves and adjusted hazard ratios and with 95% confidence intervals were generated to compare untreated and treated influenza exposure groups.

Negative Control Outcome Analysis

A negative control outcome is an analytical endpoint that is not expected to be associated with the exposure of interest.15,16 An observed association found in negative control analyses may indicate unmeasured confounding or bias. In this study, a negative control outcome would be an event resulting in hospitalization not associated with oseltamivir. Trauma or trauma-related events, assuming influenza or oseltamivir are associated with neuropsychiatric events, would not be an appropriate negative control outcome, as trauma may result from self-inflicted or abnormal behavior. There are few indications for hospitalization in children that would not be altered by the presence of an influenza infection but there are indications for hospitalization that may not be associated with oseltamivir. For

© 2025 Antoon JW et al. *JAMA Neurology*.

example, an association between influenza and appendicitis has been reported.[15,16] The proposed mechanism for influenza associated appendicitis is viral-induced lymphoid hyperplasia resulting in appendiceal lumen obstruction.[15] Nevertheless, pooled data from Phase III clinical trials in children report no difference in lymphadenopathy between children treated with oseltamivir[17] compared to those without oseltamivir exposure, suggesting that oseltamivir does not alter the potential risk of influenza induced lymphoid hyperplasia. Assuming risk of lymphadenopathy is similar across treated and untreated individuals, and that oseltamivir should not alter the frequency of appendicitis hospitalizations, we used hospitalization for appendicitis as a negative-control outcome to assess for potential bias in our study. Incidence rates of hospitalization with appendicitis were compared using multivariable Poisson regression while accounting for the study covariates, as in the main analysis.

© 2025 Antoon JW et al. *JAMA Neurology*.

eTable 1. Serious Neuropsychiatric Event Identification Algorithm

1. Primary hospital discharge diagnosis of a neuropsychiatric event<br><br>OR<br><br>2. Secondary hospital discharge diagnosis of a neuropsychiatric event with exclusion of the following primary diagnoses:<br>a) Surgical APR-DRG cases<br>b) Hematological malignancies, radiation, and chemotherapy major diagnostic category cases

© 2025 Antoon JW et al. *JAMA Neurology*.

eTable 2. Censoring Events Stratified by Exposure Group

Censoring Event	Total	No exposure	Untreated influenza	Treated influenza	Post-treatment period	Influenza prophylaxis
Loss of enrollment, n (%)	33,938 (100)	33,522 (98.8)	137 (0.4)	122 (0.3)	105 (0.4)	52 (0.2)
Age >17, n (%)*	64,463 (100)	64,290 (98.7)	34 (0.1)	32 (0.1)	42 (0.1)	65 (0.1)
Death, n (%)	31 (100)	30 (96.8)	N <11	N <11	N <11	N <11
Serious neuropsychiatric event (outcome), n (%)	1,230 (100)	1,138 (92.5)	42 (3.4)	26 (2.1)	18 (1.5)	N <11
End of study, person-weeks, n (%)	592,633 (100)	587,066 (99.1)	1,870 (0.3)	1,528 (0.3)	1,424 (0.2)	745 (0.1)

Note: inclusion criteria included age<18 years. However, an individual entering the study at a younger age and followed for multiple influenza seasons may turn 18 years of age during the study. Included individuals were censored once they reached 18 years of age.

© 2025 Antoon JW et al. *JAMA Neurology*.

eTable 3. Frequency Distribution of Neuropsychiatric Events by Category

Category	Total N, (%)	No Exposure N, (%)	Influenza and/or Oseltamivir Exposure Groups N, (%)
Any	1230 (100)	1139 (100)	91 (100)
Suicidal or self harm behaviors	420 (34.2)	407 (35.8)	13 (14.5)
Mood disorders[1]	446 (36.3)	427 (37.5)	19 (20.8)
Psychosis/hallucination	32 (2.6)	31 (2.7)	<11
Altered mental status[2]	50 (4.1)	47 (4.1)	<11
Ataxia/movement disorder[3]	11 (0.01)	<11	<11
Encephalitis	<11	0 (0.0)	<11
Seizures	168 (13.7)	137 (12.0)	31 (34.1)
Dizziness	23 (1.9)	16 (1.4)	<11
Headache	63 (0.05)	53 (4.7)	<11
Sleeping sisorders	11 (0.01)	11 (1.0)	0 (0.0)
Visions changes	<11	<11	<11

[1]Includes anxiety and stress disorders
[2]Includes paranoia, encephalopathy, delirium, and confusion
[3]Includes medication induced movement disorders.
Note: Reporting of cells with values <11 was restricted to ensure confidentiality

© 2025 Antoon JW et al. *JAMA Neurology*.

eTable 4. Incidence-Rate Ratios Examining Alternate Exposure Definitions

Exposure	Person-weeks	Serious Neuropsychiatric Events	Adjusted Incidence Rate Ratio (95% CI)
	Number		
1. Defining treated influenza as influenza diagnosis and oseltamivir days supply of ≤ 5			
Untreated influenza	70,170	42	Reference
Treated influenza	61,394	26	0.62 (0.38, 1.02)
Post-treatment period	63,303	18	0.42 (0.24, 0.74)
Influenza prophylaxis	43,789	<11	0.08 (0.03 0.26)
No exposure	19,444,929	1138	0.08 (0.06, 0.11)
2. Defining influenza prophylaxis as no influenza diagnosis and oseltamivir days supply of ≥ 7			
Untreated influenza	70,170	42	Reference
Treated influenza	94,010	32	0.47 (0.30, 0.75)
Post-treatment period	633,303	18	0.44 (0.25, 0.76)
Influenza prophylaxis	15,909	0	NA
No exposure	19,444,929	1138	0.08 (0.06, 0.10)
3. Combining Oseltamivir Treatment and Post-Treatment Period Groups			
Untreated influenza	70,164	42	Reference
Treated influenza	138,123	44	0.48 (0.31, 0.74)
Influenza prophylaxis	35,092	<11	0.10 (0.30, 0.32)
No exposure	19,444,766	1,138	0.08 (0.06, 0.11)
4. Combining Oseltamivir Exposed Individuals (Treated influenza and Influenza prophylaxis) Groups			
Untreated influenza	70,164	42	Reference
Treated influenza	109,914	32	0.36 (0.23, 0.60)
Post-treatment period	63,300	18	0.42 (0.24, 0.74)
No exposure	19,444,766	1,138	0.08 (0.06, 0.11)

Note: Cells with values <11 are not displayed to ensure individual confidentiality

© 2025 Antoon JW et al. *JAMA Neurology*.

eTable 5. Incidence-Rate Ratios of Ad-Hoc Sub-Analysis Obtaining Estimates for Alternative Outcome Definitions

		Serious neurologic event only		*Serious psychiatric events only*		*Primary neuropsychiatric diagnosis only*		*Primary diagnosis or secondary diagnosis with primary diagnosis of influenza*		*Excluding events of unclear significance**	
**Exposure**	**Person-weeks**	**Events**	**aIRR (95% CI)**	**Events**	**aIRR (95% CI)**	**Events**	**aIRR (95% CI)**	**Events**	**aIRR (95% CI)**	**Events**	**aIRR (95% CI)**
Untreated influenza	70,146	31	Reference	11	Reference	16	Reference	28	Reference	30	Reference
Treated influenza	74,807	16	0.45 (0.25, 0.83)	<11	0.80 (0.34, 1.88)	12	0.69 (0.32, 1.47)	17	0.53 (0.28, 0.98)	22	0.64 (0.37, 1.12)
Post-treatment period	63,287	<11	0.26 (0.12, 0.58)	<11	0.93 (0.40, 2.19)	14	0.86 (0.41, 1.80)	14	0.49 (0.25, 0.95)	15	0.51, 0.27, 0.95)
Influenza Prophylaxis	35,084	<11	0.12 (0.04, 0.41)	<11	0.20 (0.05, 0.95)	<11	0.19 (0.15 0.85)	<11	0.10 (0.03, 0.46)	<11	0.19 (0.07, 0.50)
No exposure	19,440,097	273	0.03 (0.02, 0.04)	865	0.22 (0.12, 0.40)	801	0.15 (0.09, 0.26)	801	0.08 (0.06, 0.13)	1056	0.10 (0.07, 0.14)

Note: Cells with values <11 are not displayed to ensure individual confidentiality
*Excludes headaches, dizziness, vision changes and sleep disturbances

© 2025 Antoon JW et al. *JAMA Neurology*.

eTable 6. Incidence-Rate Ratios When Excluding Race or Ethnicity From Statistical Model

Exposure	Person-weeks	Serious Neuropsychiatric Events	Adjusted Incidence Rate Ratio (95% CI)
	Number		
Untreated influenza	70,164	42	Reference
Treated influenza	74,823	26	0.54 (0.33, 0.88)
Post-treatment period	63,300	18	0.42 (0.24, 0.74)
Influenza prophylaxis	245,641	<11	0.10 (0.03, 0.32)
No exposure	19,444,766	1,138	0.08 (0.05, 0.10)

Note: Cells with values <11 are not displayed to ensure individual confidentiality

© 2025 Antoon JW et al. *JAMA Neurology*.

eTable 7. Incidence-Rate Ratios for Negative Control Outcome

Exposure	Person-weeks	Appendicitis Hospitalizations	Adjusted Incidence Rate Ratio (95% CI)
	Number		
Untreated influenza	70,623	<11	Reference
Treated influenza	75,358	<11	0.99 (0.39, 2.51)
Post-treatment period	63,743	<11	0.91 (0.34, 2.44)
Influenza prophylaxis	32,503	<11	0.50 (0.11, 2.32)
No exposure	18,527,306	659	0.27 (0.14, 0.53)

Note: Cells with values <11 are not displayed to ensure individual confidentiality. There were no events during the influenza prophylaxis period in which to generate an IRR.

© 2025 Antoon JW et al. *JAMA Neurology*.

eFigure 1. General Overview of Study Design and Measurements

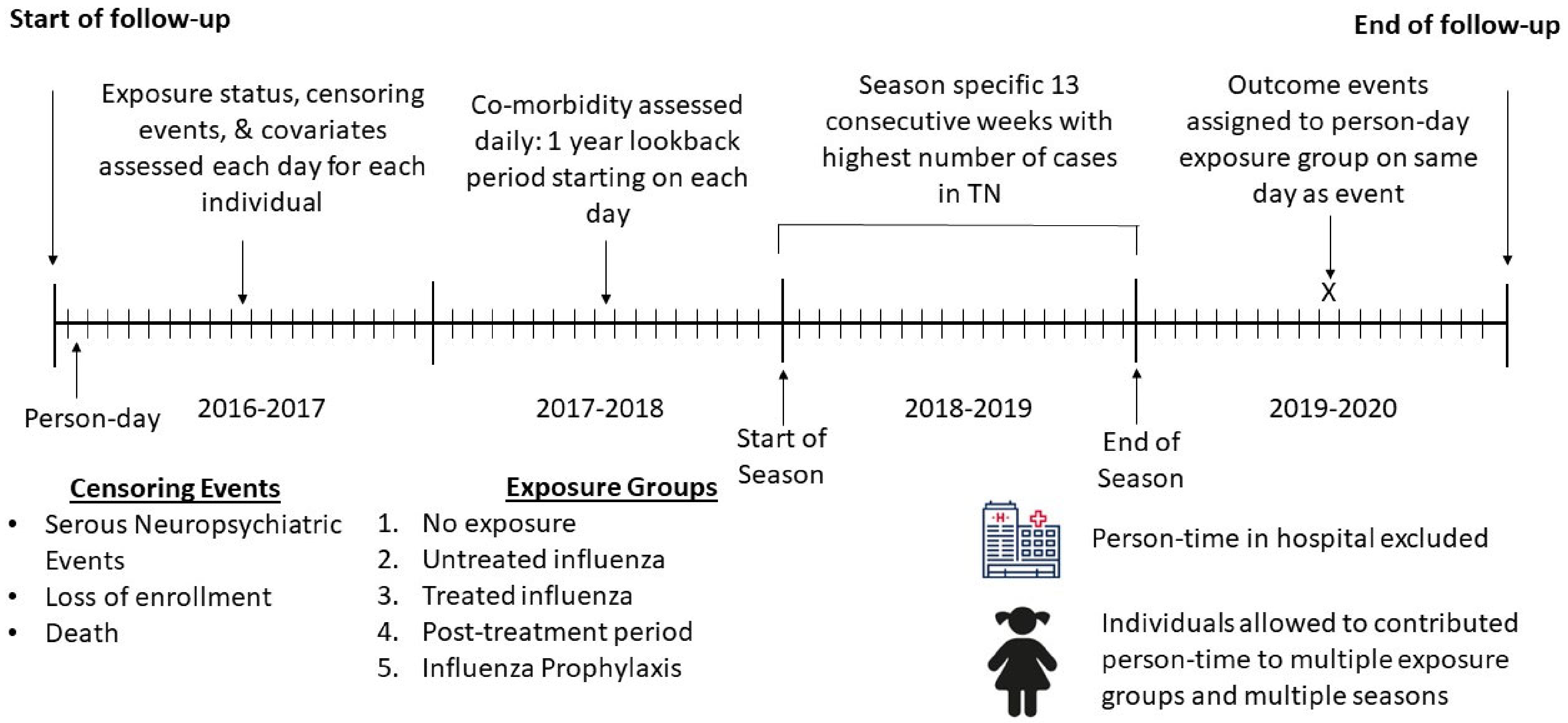

© 2025 Antoon JW et al. *JAMA Neurology*.

eFigure 2. Person-Time Assignment Examples

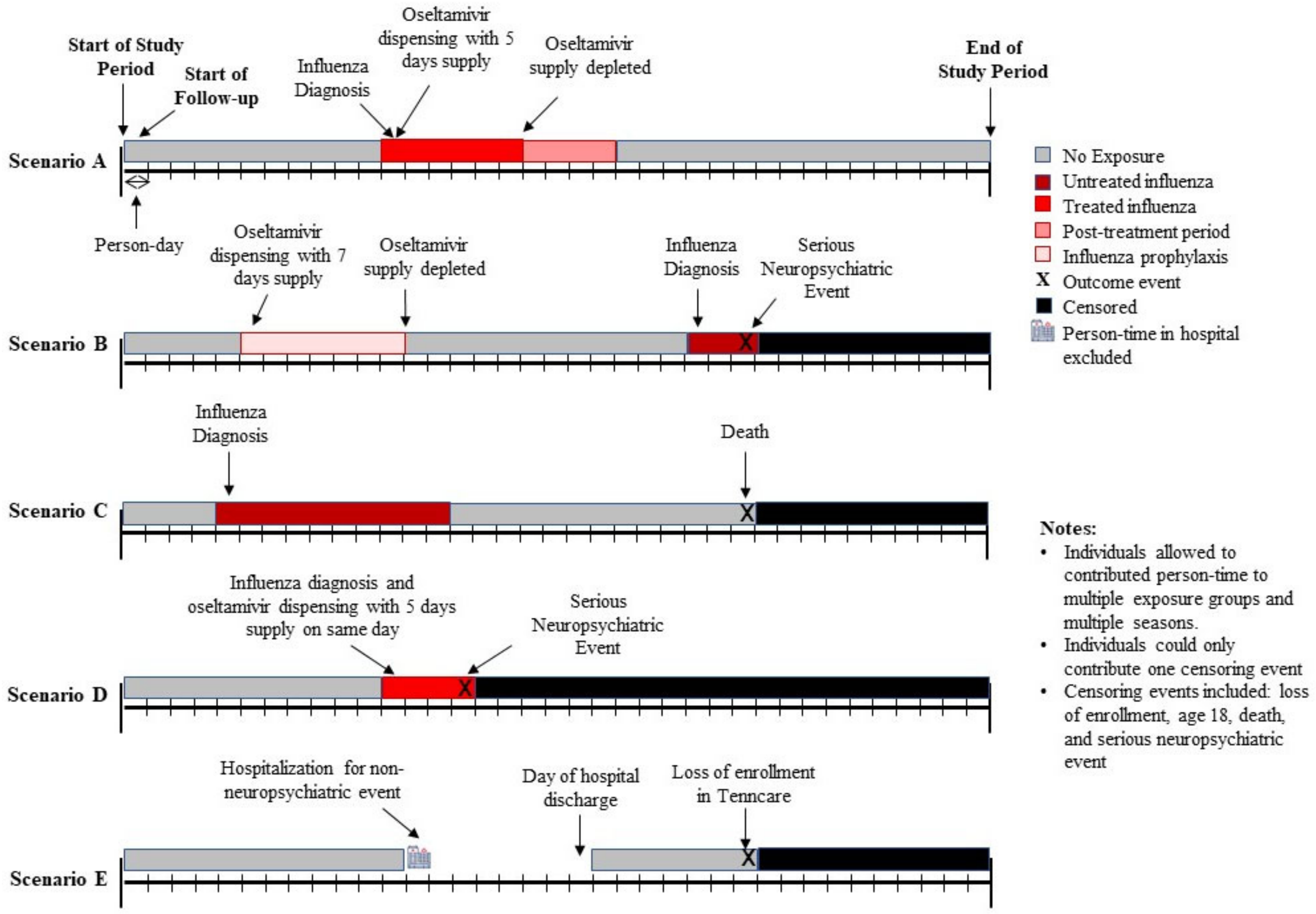

© 2025 Antoon JW et al. *JAMA Neurology*.

Each scenario is an example of the study period incorporating multiple influenza seasons to demonstrate person-time assignment at the person-day level. Spaces between each tick on the x-axis represent a single person-day. In **Scenario A**, the individual does not have a censoring event and every person-day is assigned to an exposure group. The individual contributes 27 person-days to the no-exposure group, 1 day to the untreated influenza group, 5 days to the treated influenza group, and 4 days to the post-treatment period. In **Scenario B**, the individual contributes 17 person-days to the no-exposure group, 3 days to the untreated influenza group, and 7 days to the influenza prophylaxis group. The individual is censored following their serious neuropsychiatric event and can no longer contribute person-time to the study. The event is assigned to the untreated influenza exposure group. In **Scenario C**, the individual contributes 17 person-days to the no-exposure group and 10 days to the untreated influenza group. The individual is censored following their out-of-hospital death and the event is assigned to the no-exposure group. In **Scenario D**, the individual contributes 11 person-days to the no-exposure group and 4 days to the treated influenza group. The individual is censored following their serious neuropsychiatric event and the event is assigned to the treated influenza exposure group. In **Scenario E**, the individual contributes 19 person-days to the no exposure group. The individual was hospitalized for 8 days, and those days are not assigned to any exposure group and not included in the study. The individual is censored following the loss of enrollment in TennCare program.

© 2025 Antoon JW et al. *JAMA Neurology*.

eFigure 3. Time Between Start of Exposure and Serious Neuropsychiatric Outcome Event

A

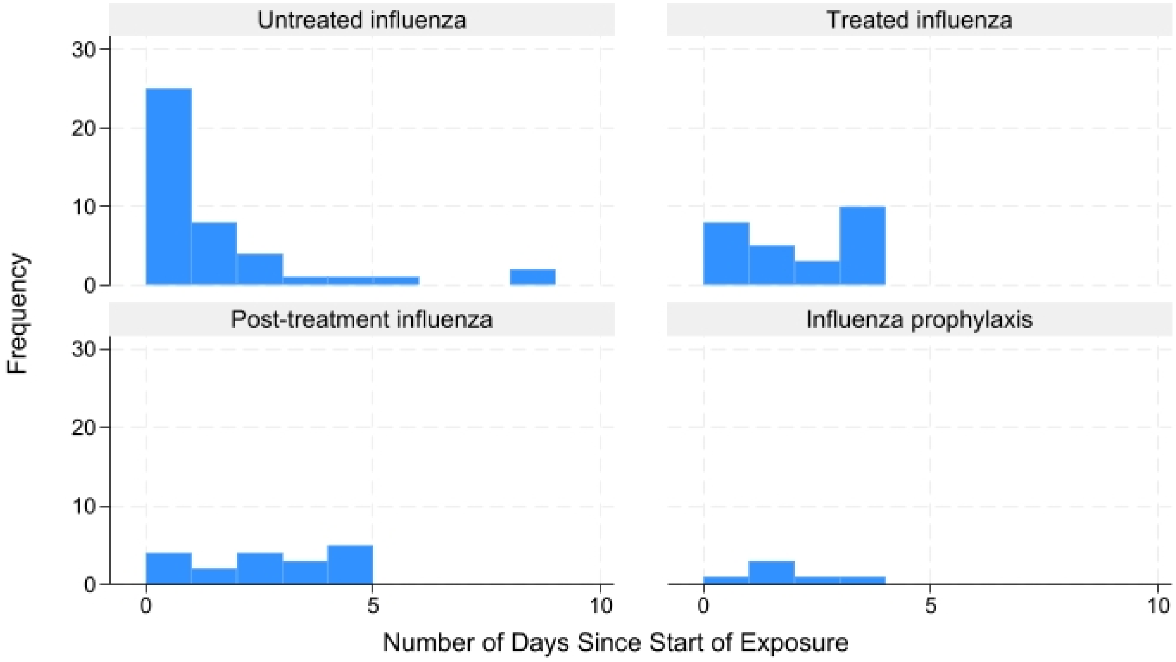

B

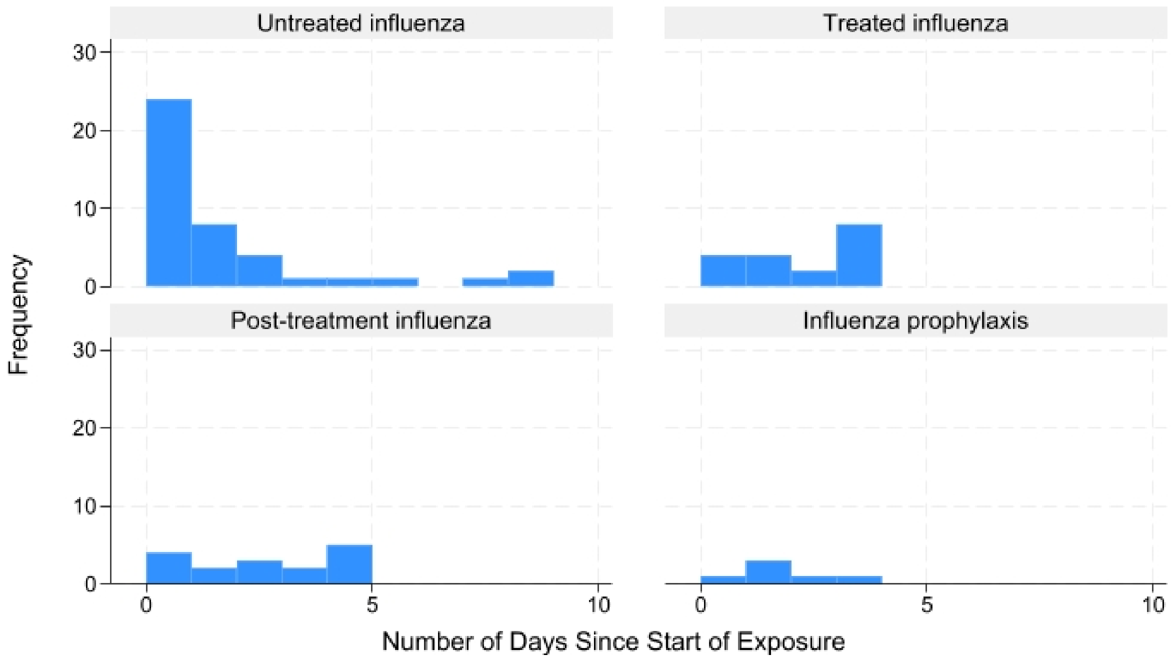

(A) Histogram of the number of days from the start of exposure to outcome event among individuals who experienced a serious neuropsychiatric event. The first day of exposure was defined as day 0. (B) Histogram of the number of days from the start of exposure to outcome event among individuals who experienced a serious neuropsychiatric event when excluding influenza episodes with oseltamivir dispensing after day of diagnosis. For untreated and treated influenza, day 0 is day of influenza diagnosis as all treated influenza episodes were dispensed oseltamivir on day of influenza diagnosis.

© 2025 Antoon JW et al. *JAMA Neurology*.

eFigure 4. E-Value Analysis

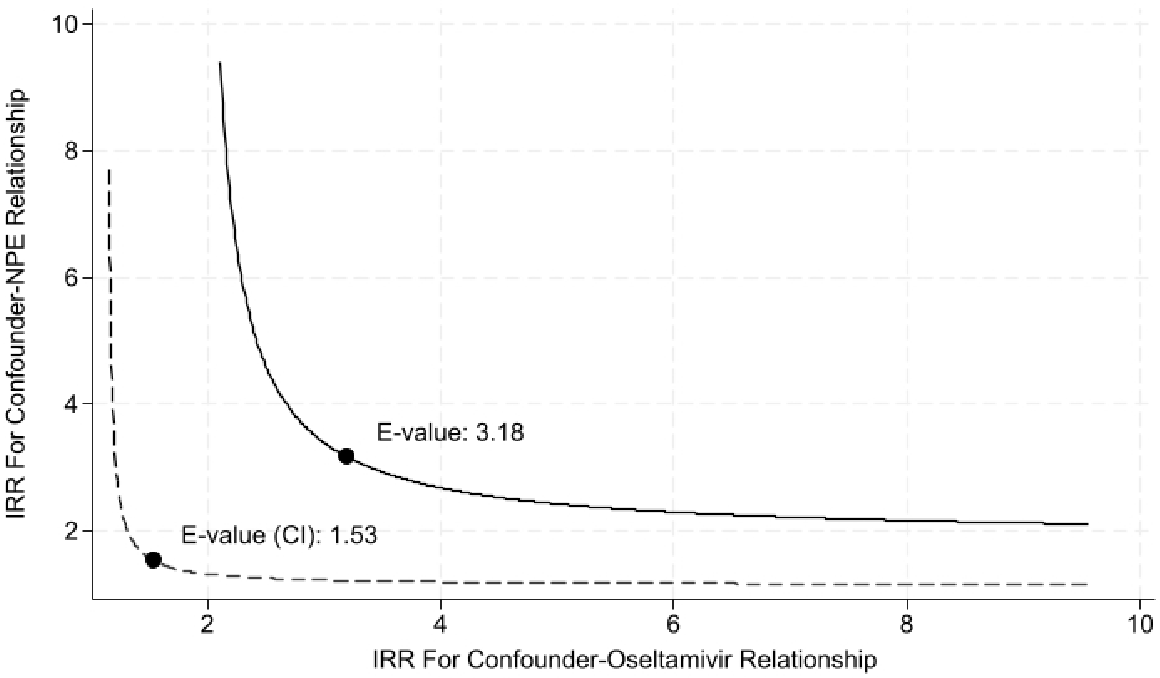

Each point along the curve defines a joint relationship between oseltamivir and serious neuropsychiatric events that could potentially explain away the estimated association. If the relationship of the unmeasured confounder and either oseltamivir or serious neuropsychiatric events is smaller than the E-value, the other must be larger, as defined by the plotted curve. The solid line represents the E-value curve for the primary point estimate. The dotted line represents the E-value curve for the upper confidence interval associated with the primary point estimate.

© 2025 Antoon JW et al. *JAMA Neurology*.

eReferences.

1. Antoon JW, Williams DJ, Bruce J, et al. Population-Based Incidence of Influenza-Associated Serious Neuropsychiatric Events in Children and Adolescents. *JAMA Pediatr*. Sep 1 2023;177(9):967-969. doi:10.1001/jamapediatrics.2023.2304
2. Antoon JW, Stopczynski T, Amarin JZ, et al. Accuracy of Influenza ICD-10 Diagnosis Codes in Identifying Influenza Illness in Children. *JAMA Netw Open*. Apr 1 2024;7(4):e248255. doi:10.1001/jamanetworkopen.2024.8255
3. Grijalva CG, Poehling KA, Edwards KM, et al. Accuracy and interpretation of rapid influenza tests in children. *Pediatrics*. Jan 2007;119(1):e6-11. doi:10.1542/peds.2006-1694
4. Leeflang MM, Rutjes AW, Reitsma JB, Hooft L, Bossuyt PM. Variation of a test's sensitivity and specificity with disease prevalence. *CMAJ*. Aug 6 2013;185(11):E537-44. doi:10.1503/cmaj.121286
5. Benack K, Nyandege A, Nonnenmacher E, et al. Validity of ICD-10-based algorithms to identify patients with influenza in inpatient and outpatient settings. *Pharmacoepidemiol Drug Saf*. Apr 2024;33(4):e5788. doi:10.1002/pds.5788
6. Poehling KA, Edwards KM, Weinberg GA, et al. The underrecognized burden of influenza in young children. *N Engl J Med*. Jul 6 2006;355(1):31-40. doi:10.1056/NEJMoa054869
7. Feudtner C, Feinstein JA, Zhong W, Hall M, Dai D. Pediatric complex chronic conditions classification system version 2: updated for ICD-10 and complex medical technology dependence and transplantation. *BMC Pediatr*. Aug 8 2014;14:199. doi:10.1186/1471-2431-14-199
8. Zima BT, Gay JC, Rodean J, et al. Classification System for International Classification of Diseases, Ninth Revision, Clinical Modification and Tenth Revision Pediatric Mental Health Disorders. *JAMA Pediatr*. Jun 1 2020;174(6):620-622. doi:10.1001/jamapediatrics.2020.0037
9. Committee On Infectious Diseases. Recommendations for Prevention and Control of Influenza in Children, 2022-2023. *Pediatrics*. Oct 1 2022;150(4)doi:10.1542/peds.2022-059275
10. Uyeki TM, Bernstein HH, Bradley JS, et al. Clinical Practice Guidelines by the Infectious Diseases Society of America: 2018 Update on Diagnosis, Treatment, Chemoprophylaxis, and Institutional Outbreak Management of Seasonal Influenzaa. *Clin Infect Dis*. Mar 5 2019;68(6):895-902. doi:10.1093/cid/ciy874
11. Grohskopf LA, Blanton LH, Ferdinands JM, et al. Prevention and Control of Seasonal Influenza with Vaccines: Recommendations of the Advisory Committee on Immunization Practices - United States, 2022-23 Influenza Season. *MMWR Recomm Rep*. Aug 26 2022;71(1):1-28. doi:10.15585/mmwr.rr7101a1
12. Antoon JW, Hall M, Feinstein JA, et al. Guideline Concordant Antiviral Treatment in Children at High-risk for Influenza Complications. *Clin Infect Dis*. Jul 22 2022;doi:10.1093/cid/ciac606
13. Antoon JW, Grijalva CG, Thurm C, et al. Factors Associated With COVID-19 Disease Severity in US Children and Adolescents. *J Hosp Med*. Oct 2021;16(10):603-610. doi:10.12788/jhm.3689
14. Antoon JW, Feinstein JA, Grijalva CG, et al. Identifying Acute Neuropsychiatric Events in Children and Adolescents. *Hosp Pediatr*. May 1 2022;12(5):e152-e160. doi:10.1542/hpeds.2021-006329
15. Soltani S, Kesheh MM, Siri G, et al. The role of viruses in human acute appendicitis: a systematic literature review. *Int J Colorectal Dis*. Apr 18 2023;38(1):102. doi:10.1007/s00384-023-04391-z

© 2025 Antoon JW et al. *JAMA Neurology*.

16. Alder AC, Fomby TB, Woodward WA, Haley RW, Sarosi G, Livingston EH. Association of viral infection and appendicitis. *Arch Surg*. Jan 2010;145(1):63-71. doi:10.1001/archsurg.2009.250
17. Roche Pharmaceuticals. Tamiflu (oseltamivir phosphate) [package insert]. U.S. Food and Drug Administration https://www.accessdata.fda.gov/drugsatfda_docs/label/2012/021087s062lbl.pdf. Revised December 2012. Accessed March 28, 2024.

© 2025 Antoon JW et al. *JAMA Neurology*.
