# Supplementary material for: Influenza With and Without Oseltamivir Treatment and Neuropsychiatric Events Among Children and Adolescents
Source: JAMA Neurol. 2025 Aug 4;82(10):1013–21. doi: 10.1001/jamaneurol.2025.1995 (PMC12322824; doi:10.1001/jamaneurol.2025.1995)
Supplement: Supplement 2. — Data Sharing Statement [file jamaneurol-e251995-s002.pdf]

Data Sharing Statement

Antoon. Influenza With and Without Oseltamivir Treatment and Neuropsychiatric Events Among Children and Adolescents. *JAMA Neurol*. Published August 04, 2025. doi:10.1001/jamaneurol.2025.1995

Data
Data available: No

Additional Information
Explanation for why data not available: Data use agreement with the TennCare program restricts sharing of individual patient level data. Access to the data can be requested through the TN Dept of Health and Division of TennCare
